# Factors Associated with Low Levels of HIV Testing among Men Who Have Sex with Men (MSM) in Brazil

**DOI:** 10.1371/journal.pone.0130445

**Published:** 2015-06-22

**Authors:** Ana Maria Brito, Carl Kendall, Ligia Kerr, Rosa Maria Salani Mota, Mark Drew Crosland Guimarães, Inês Dourado, Adriana A. Pinho, Adele Schwartz Benzaken, Sandra Brignol, Arthur L. Reingold

**Affiliations:** 1 Centro de Pesquisas Aggeu Magalhães, FIOCRUZ, Recife, PE, Brazil; 2 Center for Global Health Equity, Tulane University School of Public Health and Tropical Medicine, New Orleans, Louisiana, United States of America; 3 Department of Community Health, Universidade Federal do Ceará, Fortaleza, CE, Brazil; 4 School of Medicine, Universidade Federal de Minas Gerais, Belo Horizonte, Brazil; 5 Institute of Collective Health, Universidade Federal da Bahia, Salvador, BA, Brazil; 6 Instituto de Comunicação e Informação Científica e Tecnológica em Saúde, Fiocruz, Rio de Janeiro, RJ, Brazil; 7 Fundação Alfredo da Mata, Manaus, AM, Brazil; 8 School of Public Health, University of California, Berkeley, California, United States of America; University of Toronto, CANADA

## Abstract

The aim of this study was to assess risk factors associated with low levels of HIV testing among MSM recruited through respondent driven sampling (RDS) in Brazil. Of 3,617 participants, 48.4% had never tested previously for HIV. A logistic model indicated that younger age, lower socioeconomic class, education, poor HIV/AIDS knowledge, no history of cruising, and having been tested during the study were characteristics independently associated with low levels of previous HIV testing. The HIV testing rate among MSM in Brazil is still low in spite of the availability of a large number services providing universal and free access to HIV/AIDS diagnosis and treatment. To respond to low utilization, the authors propose a higher priority for testing for key populations such as MSM, expanded education, expanding testing sites and a welcoming and nonjudgmental environment in health services.

## Introduction

Despite preventive measures widely available to reduce the spread of HIV infection and other sexually transmitted infections (STIs) among men who have sex with men (MSM), this population continues to be disproportionately affected by HIV in countries with higher [1–4] and medium to low Gross Domestic Product (GDP)[5, 6]. In the United States (U.S.), in 2010, MSM accounted for 78% of newly diagnosed cases of HIV among men 13 years of age and older, and constituted 61% of all those testing positive receiving a diagnosis of HIV (CDC, 2011). Also in the U.S., in a sample of 6,672 men of unknown serological status undergoing testing, the risk of men reporting male-male sex was ten times greater than that of men who did not have sex with men [7]. In Brazil, Barbosa et al. [8] found an AIDS incidence rate thirteen times higher among MSM than among heterosexual men. A study carried out in the Southeast region [9] showed that 7% of MSM were infected with HIV while Kerr et al. [10] found a prevalence of HIV among MSM of 5.2 to 23.7% in ten Brazilian cities, with an overall prevalence (14.2%) two to three times greater than that estimated for female sex workers and drug users in these same cities [11].

HIV infection risk among MSM involves multiple factors, including lack of knowledge of serological status [12, 13]. The promotion of HIV testing has been a key element in HIV transmission prevention strategies; especially among populations most at risk for STIs, such as MSM, sex workers and drug users. Studies have shown that many MSM, including gays and bisexuals, are unaware of their serological status, and many likewise have no knowledge of the serological status of their partner [1, 4, 14]. Studies conducted in developed countries show that the proportion of MSM never testing is generally below 30% [3, 15–19], while in some countries in Latin America, Africa and Asia the figure for never testing is 40% or higher [20–25].

Even though Brazil has one of the most successful AIDS programs in the world, including universal free access to HIV testing within the public health system, there are no population-based studies of testing among MSM. Although there has been an increase in the proportion testing from 20.2%, in 1998, to 33.6%, in 2005, men with less schooling and those who live in less socio-economically developed regions of the country were less likely to be tested [26]. Data from the Brazilian Ministry of Health indicates that among the estimated 630,000 HIV positive population nationwide, 255,000 have never been tested [27].

Characteristics associated with not testing reported in several studies in the United States include a low level of knowledge of the risk of individual infection by HIV, fear of knowing the result of the test and youth [28–31]. Non-white MSM are more likely to not test for HIV compared to white men [31]. Lack of access to free testing and other structural barriers, such as lack of transportation, not knowing where to test, cultural barriers, and concerns regarding the confidentiality of the HIV test have also been cited as reasons for not testing in China [25]. On the other hand, factors associated with testing at least once includes having exclusively male partners, having had a diagnosis of an STI, having more sexual partners, having a gay or homosexual identity, and being aware of the risk of infection [28, 32, 33].

Lack of knowledge of serological status has negative consequences in the control of the HIV epidemic, from the point of view of both prevention and treatment. Early initiation of antiretroviral therapy—a potential consequence of enhanced testing—has shown to reduce rates of sexual transmission of HIV-1 and clinical events, indicating both individual and public health benefits from such therapy [34]. It is estimated that new HIV infections could be reduced by 30% per year, if all infected individuals were aware of their serological status and acted appropriately on it [35]. In view of the paucity of Brazilian studies regarding HIV testing among MSM, this study aims to estimate the magnitude of never testing for HIV and factors associated with not testing among MSM in Brazil. We anticipate that lower socioeconomic status and risk behaviors are associated with lack of testing.

## Methods

### Type of study, population and data collection procedures

Data were collected as part of a cross-sectional National Multicenter Study among MSM conducted in ten Brazilian cities between October 2008 and June 2009. The population included men reporting male-male sex, and excluded transwomen (*travesti*). The study was approved by the Brazil National Ethical Research Committee (CONEP # 14494). Participants signed two separate consent forms: one to participate in the questionnaire component of the survey, and one to agree to test and to report results for use in the study.

Participants were 18 years of age or older and resident in Manaus, Recife, Salvador, Belo Horizonte, Rio de Janeiro, Santos, Curitiba, Itajaí, Brasília or Campo Grande. The cities were selected by the Ministry of Health, who funded this study. There are multiple reasons for choosing these cities, but one reason was the assumption of large populations of MSM living in these cities. Additionally, each region of the country was represented by at least one city. Participants were eligible if they reported at least one sexual contact with another man in the twelve months prior to the interview. The sample was recruited utilizing Respondent Driven Sampling (RDS)[36, 37] and main findings of the overall study have been reported in Kerr et al. [10]. RDS is a chain link sampling method that begins with a convenience sample of members of the target population called ‘seeds’. Seeds recruit a prespecified number of recruits. These respondents in turn recruit new participants. With some assumptions, this method produces recruitment chains that, when long enough, are much less dependent on the purposively selected seeds. The personal social network size of respondents is used to weight the influence of network size and differential recruitment. RDS is widely used as an HIV surveillance-sampling method [38] and has been recommended by the Centers for Disease Control and Prevention [39]and by Brazil Department of STIs, AIDS and Viral Hepatitis [40] for hidden, hard-to-reach, and HIV high-risk populations. Network size, waves, homophily and wave to achieve equilibrium by city can be found in Tables A and B in S1 Appendix. The dataset can be found in S2 Appendix. The file contains a workbook with the data formatted for RDSAT and a data dictionary.

A face-to-face questionnaire that included questions concerning socio-demographic factors, sexual behavior, drug use, social context, network of contacts, healthcare, access to condoms, previous HIV and syphilis testing and information on STIs was applied. The interview was followed by separately consented rapid tests for HIV and syphilis. A total of 3,959 men over 18 years of age were recruited in the 10 sites (range 274 in Belo Horizonte to 848 in Manaus). Time of recruitment varied from 20 to 34 weeks. This study was initiated with six seeds representing different age and social class in each municipality identified in formative research conducted in each city. Additional seeds were added if recruitment slowed. A total of 140 seeds were utilized across the sites, varying from 7 seeds in Campo Grande to 32 in Curitiba. Each participant received three coupons to invite participants to the study. This process was repeated until the study reached the desired sample size. Participants received a primary incentive of R$ (Brazilian Real) 15.00 (US$ 10.00) and an incentive of R$(Brazilian Real) 10.00 (US$ 6.67) for each of their recruits who completed the survey. Sites selected were all health units of City Health Departments. Surveys included 12–20 waves of data collection in each site. A single participant refused to participate and 3–50 recruits, depending on the city, were considered ineligible upon arrival at the sites. Analysis used RDSAT 5.6 to adjust values for size of social network and recruitment. Readers with additional interest in RDS are referred to a previous publication [10] and to the on-line appendix to this paper: Details of RDS in the Multicenter study 2008–2009.

### Outcome and exposure measures

For the purpose of this analysis, the outcome of interest was lifetime HIV testing prior to the study. Those respondents who reported never testing prior to the study were compared to those with at least one prior HIV test. The explanatory variables were arranged in three modules. Module 1 included age, race, marital status, schooling, and current employment. Module 2 included variables related to sexual orientation, sexual identity, family acceptance of sexual status, sex of the first sexual partner, age at sexual debut, number of casual partners in the past year, number of sexual partners in the past six months, consumption of alcoholic beverages, use of crack and consumption of marijuana in the past six months. Module 3 consisted of characteristics related to knowledge of HIV transmission and testing: self-perception of risk, correct knowledge of transmission, and testing during the interview. Knowledge of transmission was assessed using nine questions and respondents were scored as low, average or high knowledge when they responded correctly to less than 4, 5 or 6, and 7 or more correct responses, respectively. Economic status was measured using standard Brazilian criteria published by the Brazilian Association of Research Companies (ABEP)[41]. The widely used criteria include wealth measures and education of household head, and are modified annually. “A” constitutes the highest social class, while “E” the lowest. We used criteria from 2008.

### Statistical Analysis

Data from each survey were weighted by the inverse probability of individual selection, proportional to the size of the social network reported by each respondent and the proportional size of the MSM population in each municipality [42]. The size of the MSM population had been previously estimated in a national survey [8].

The analysis of association with the outcome of interest used Pearson’s chi-squared test with the level of significance set to 0.05. Weighted estimates and prevalence ratio with their respective confidence intervals set to 95% were obtained using the complex sampling model for stratified sampling (stratum = city) using Stata 11.0. The magnitude of the associations was calculated using the weighted odds ratio (WOR) with a confidence interval of 95%. Weighted multivariate logistic regression was used to assess the effect of potentially explanatory independent variables. The multivariate logistic regression modeling only included variables that were found significant at the level of p<0.10. While p<0.25 is frequently recommended for variable selection, at this level a very large number of variables are selected, and many demonstrate multicollinearity. Only those variables with a statistical significance level of p <0.05 were retained in the final model.

## Results

### Characteristics of participants

Of the 3,859 participants recruited, 3,617 (93.7%) provided information about prior testing. Of these, 1,715 (48.4%; 95% CI, 45.1–51.8%) had never been previously tested for HIV. Participants were mostly young, of mixed race (57.6%), single (84.4%), with a low level of education, unemployed, and living on a monthly income of less than one Brazilian minimum wage (R$ 500 = US$275). Most self-identified themselves as gay or homosexual (63.1%), but more than half reported feeling attracted to both sexes. Half of the participants reported a woman as their first sexual partner and most used a condom (69.7%) during their sexual debut. About half of the participants reported discrimination from their family because of their sexual orientation and most had a medium to high level of knowledge of HIV transmission. At the same time, a high proportion (69.0%) considered their own chance of becoming infected as low or zero. Most of the participants reported more than three casual partners in the past year; more than half reported using a condom during all sexual relations with commercial male partners during the past year, while less than 40% reported using a condom with casual female partners. Half of the participants reported having been subjected to some kind of sexual violence.

### Univariate and multivariate analysis

Univariate analysis is shown in Table 1. The proportion of participants who never tested was significantly higher among younger, non-white men with lower levels of schooling, from lower social classes, and who were unemployed or living on a low income. Those who reported attraction towards men and women, had a bisexual and/or heterosexual identity, had a woman as their first sexual partner, were older than fifteen years at their sexual debut, had fewer sexual partners in the past year, not having sought sexual partners at cruising sites (bars, discos, saunas, pornographic movie theatres) in the past year, not having been exposed to sexual violence or not having been forced to have male-male sexual relations against their will were found more likely to have never been tested. Of the Module 3 variables, those that were significantly associated with never testing for HIV were low levels of knowledge of the modes of transmission of HIV, not feeling at risk of infection, and agreeing to be tested during the study.

**Table 1:** Univariate analysis of never testing for HIV among MSM in Brazil, 2009.

Group / variables	Total	No-HIV	Testing	Odds Ratio (95%%CI)	p-value
	(N = 3,617)	n(crude)	%(weighted)		
**SOCIO-DEMOGRAPHIC**					
***Age (years)*:**					
≤ 20	904	642	71.6	4.05 (2.52; 6.52)	<0.001
21–29	1552	700	46.7	1.4 (0.92; 2.13)	
30–39	734	244	40.6	1.09 (0.67; 1.78)	
≥ 40	413	124	38.4	1.0	
***Skin Color/Race*:**					
Non-white	2625	1354	52.0	1.74 (1.26; 2.42)	0.001
White	990	361	38.2	1.0	
***Marital status*:**					
Single/Widow/Divorced	3011	1476	49.7	1.38 (0.94; 2.0)	0.092
Married (M or F partner)	605	239	41.8	1.0	
***Schooling (years)*:**					
**≤ 8**	1068	640	58.9	3.84 (2.64; 5.59)	<0.001
**09–12**	1594	819	48.9	2.57 (1.81; 3.66)	
**≥ 13**	940	250	27.1	1.0	
***Social class (criteria Brazil)***					
**A/B**	1007	312	26.4	1.0	<0.001
**C**	1815	951	50.9	2.89 (2.11; 3.97)	
**D/E**	794	451	59.4	4.08 (2.81; 5.94)	
***Individual income (in Brazilian Real*** ^***1***^ ***)***					
None	732	486	58.4	6.18 (3.2; 11.94)	<0.001
R$ 100–< R$ 500	1042	591	58.9	6.29 (3.44; 11.50)	
R$ 500–999	1122	481	46.3	3.79 (2.06; 7.00)	
R$ 1000–1999	463	116	21.9	1.23 (0.62; 2.46)	
≥ R$ 2000	231	40	18.5	1.0	
***Currently working***					
Yes	1454	567	37.0	1.0	<0.001
No	2032	1071	53.6	1.96 (1.49; 2.59)	
**SEXUAL AND SOCIAL BEHAVIOR**					
***Sexual attraction to***					
Only Men	1747	627	36.7	1.0	<0.001
Both (women and men)	1870	1088	56.9	2.28 (1.72; 3.0)	
***Use of marijuana (Last 6 months)***					
No	2528	1155	47.4	1.0	0.341
≤ once a week	603	288	47.7	1.01 (0.69; 1.46)	
≥ twice a week	474	266	54.8	1.34 (0.89; 2.01)	
***Suffered any violence (verbal / physical/sexual)***					
No	1693	888	55.6	1.75 (1.34; 2.3)	<0.001
Yes	1918	825	41.6	1.0	
***Suffered verbal abuse due to sexual orientation***					
No	1948	1002	54.0	1.66 (1.26; 2.18)	<0.001
Yes	1663	711	41.4	1.0	
***Physical violence due to sexual orientation***					
No	3064	1475	49.3	1.23 (0.85; 1.78)	0.264
Yes	547	238	44.1	1.0	
***Suffered sexual violence***					
No	3028	1505	51.1	1.97 (1.34; 2.88)	<0.001
Yes	583	208	34.7	1.0	
**KNOWLEDGE AND RISK PERCEPTION**					
***Knowledge about HIV transmission and HIV test***					
Low (0–4)	415	306	73.3	5.07 (3.1; 8.29)	<0.001
Medium (5–6)	1759	888	51.5	1.95 (1.45; 2.64)	
High (7–9)	1435	515	35.2	1.0	
***HIV risk self-perception***					
No risk	1020	580	58.7	1.62 (1.16; 2.26)	0.021
Low risk	1475	698	46.7	1.0	
High risk	969	437	51.0	1.19 (0.85; 1.67)	
***HIV test during the study***					
No	420	106	23.3	1.0	<0.001
Yes	3197	1609	50.4	3.34 (2.2; 5.1)	

The results of the weighted multivariate logistic regression are presented in Table 2. The number of valid observations in the multidimensional model was 3,591. The final adjusted regression model can generate estimates for the outcome variable. Comparing the model to the actual outcomes, the adjusted model was 71.1% accurate, with a sensitivity of 70.4% and a specificity of 71.7%. The final model shows that not having tested for HIV test was associated (p < 0.05) with lower age (OR = 5.29, 1.64 and 1.2 for those less than <20, 20–29 and 30–39 years old, respectively); lower social class (OR = 2.87 and 2.21 for classes D/E and C, respectively); lower level of education (OR = 1.61 and 1.30 for less than 8 years and 9 to 12 years, respectively); feeling sexual attraction for both men and women (OR = 1.74); having sought out sexual partners at cruising locations (OR = 1.86); not having suffered sexual violence (OR = 1.87); lower level of knowledge about HIV transmission and HIV testing (OR = 2.78 and 1.47 for low and medium levels, respectively); and agreeing to test during the study (OR = 3.17).

**Table 2:** Multivariate analysis (final model) of never testing for HIV among MSM in Brazil, 2009.

Variables	Odds ratio	95% IC	p-value
***Age (years old)***			
≤ 20	5.29	(3.21; 8.71)	<0.001
21–29	1.64	(1.05; 2.56)	
30–39	1.20	(0.72; 2.00)	
≥ 40	1.0		
***Schooling (years)***			
≤ 8	1.61	(1.01; 2.57)	0.05
9–12	1.30	(0.87; 1.95)	
≥ 13	1.0		
***Social class***			
A/B (higher)	1.0		<0.001
C	2.88	(1.77; 4.67)	
D/E (lower)	2.22	(1.51; 3.28)	
***Sexual attraction to***			
Men only	1.0		<0.001
Women and men	1.74	(1.29; 2.35)	
***Cruising (last month)***			
No	1.00		<0.001
Yes	1.87	(1.35; 2.58)	
***Suffered Sexual violence***			
No	1.87	(1.24; 2.82)	0.003
Yes	1.0		
***Knowledge about HIV transmission and HIV testing***			
None/low (0–4)	2.79	(1.61; 4.81)	0.001
Medium (5–6)	1.47	(1.07; 2.04)	
High (7–9)	1.0		
***HIV test during the study***			
No	1.0		<0.001
Yes	3.17	(2.00; 5.02)	

## Discussion

Our results demonstrate that a high proportion (48.4%; 95% CI: 45.1–51.8) of Brazilian MSM in the study had never tested for HIV prior to the study. Rates are higher, though, than in countries where the issues of stigma, discrimination and, in some cases, the criminalization of homosexuality pose significant obstacles to testing, including several African countries [43], [44], [6] and other Latin American and Asian countries [45–48]. In most of these countries, researchers argue that lack of behavior change communication and other programs to promote prevention and early treatment, low quality of programs directed at and poor coverage for MSM, and limited surveillance systems also contribute to a low testing rate[49]. Inversely, in countries that have adopted strategies to promote testing, such as Cuba [50], Italy, Australia and Canada [18], the United Kingdom [16] and the United States [2, 3], the proportion of MSM never testing is low.

Despite differences in the magnitude of never testing, compared with countries with epidemiological patterns similar to Brazil, factors statistically associated with the lack of HIV testing are similar to those reported in the literature [2, 3, 16, 18, 50]. The higher proportion of never testing among younger age groups is corroborated by several authors [30, 31, 51]. Our results are also similar to other studies with regard to knowledge of the modes of HIV transmission: the lower the level of knowledge concerning HIV prevention the greater the likelihood that MSM do not test [51, 52]. Studies have also shown that a low level of perception of the risk of HIV infection is associated with not testing [25].

The finding that individuals who had never tested prior to the study were three times more likely to voluntarily take an HIV test compared to those MSM who admitted having tested as least once may indicate a low refusal rate for voluntary testing when the HIV test is provided in the circumstances of a special study. Some studies have shown that not knowing where to test and fear of the consequences of testing positive are two of the main reasons cited for not testing [16, 20, 23, 25]. On the other hand, it is likely that people who have recently tested—especially if they are seropositive—may not wish to repeat the experience. In terms of sexual behavior, an association was found between “feeling sexually attracted towards men and women” and never having taken an HIV test. These individuals are more than twice as likely not to have been tested as compared to those who are attracted only to men. This may be related to a higher level of internalization of negative views regarding homosexuality among bisexuals, which, as shown by other studies is associated with lower access to STD services and HIV testing [53]. Inversely, the finding that there is an association between sexual violence and testing may suggest that sexual violence induces individuals to seek HIV testing.

There is a worldwide tendency towards increased HIV testing, especially among the populations most at risk of HIV infection. In most developed countries, since the mid-1990s, one of the main components of HIV-prevention policy has been to increase the number of HIV-infected individuals who are aware of their serostatus [16]. The World Health Organization (2011) recommends that all MSM take at least one HIV test per year, and that those MSM who have multiple or anonymous partners, those who take illegal drugs, or those whose partners participate in such activities, should test with a greater frequency of every 3 or 6 months.

In Brazil, it has been more than twenty years since the implementation of a national system of Voluntary Counseling and Testing (VCT) in clinics and specialized centers. These centers are designed to promote access to free, confidential, anonymous and rapid HIV testing, especially for populations at greater risk of exposure to HIV (MSM, sex workers and drugs users) and, in collaboration with other health services, to refer individuals receiving a positive diagnosis. However, studies have shown that VCT have significant limitations in terms of quality of diagnostic services and in promoting prevention measures [54, 55], and the performance of individual VCTs vary greatly. These studies have identified difficulties in linking VCT with the broader health care network, including shortcomings in referral and counter-referral and low productivity, with few HIV tests being conducted in relation to the installed capacity, especially among MSM. The findings of the present study regarding the high percentage of MSM not testing corroborate this evaluation of the performance of the VCT.

In view of estimates that the incidence of new infections continues to be greater among the most vulnerable groups and the fact that members of these groups are probably unaware of their HIV status, the Brazilian Ministry of Health launched the “*Fique sabendo*+” (Be Aware +) campaign in 2008, attempting to increase HIV testing (51). Artists and other opinion leaders are key figures in the campaign to encourage regular testing and to reduce prejudice towards persons living with HIV/AIDS.

Although broader provision of testing is one of the priorities of the Brazilian National Program to combat AIDS, and efforts to integrate testing and promotion of testing at all levels of the health service have been effective [56], efforts to date do not appear to have overcome resistance to testing. Although efforts to promote testing should be redoubled, it is disappointing to note that the recent National Plan to combat the AIDS epidemic and STDs among Gays, other MSM and Transwomen, does not mention testing as a prevention strategy in any of its nine guidelines.

There are limitations to our study: the cross-sectional research design does not allow inference of causality, and RDS and especially RDS in ten Brazilian cities, require some strong assumptions for interpretation, including combining values from the 10 cities, adjusting for estimated proportions of MSM, and estimation methods to adjust for biases in RDS, a chain-link sampling method. Another potential limitation is that the study is dated, based on a survey conducted in 2008–2009, and there have been changes to expand testing in Brazil since that time. However, we still feel that publication at this time is warranted. Many HIV-related indicators point in the wrong direction: HIV seropositivity is increasing among conscripts to the military and among MSM, and AIDS cases are increasing among young MSM as well [11]. This may be related to changing policies introduced by the government under pressure from religious leaders that prohibited prevention programs from addressing MSM and female sex workers, and defunding the NGOs that played such as important role in Brazil’s response to AIDS in these key populations.

Despite these limitations, in the aggregate or by city, the proportion of MSM who never tested is striking. Testing levels are lowest among those who are younger, of lower socioeconomic class, have low levels of education and have poor HIV/AIDS knowledge. Efforts to overcome barriers to testing are not reaching this population as they should. In view of the existing large network of health services providing universal and free access to treatment in Brazil, this low level is difficult to understand. Underutilization may be due to the number and availability of providers, including the policy of limiting testing to VCT centers. Limiting testing to VCT or selected health services, without considering alternative sites such as NGOs, does not appear to be a reasonable public health policy given the receptiveness to testing in our study. While access for testing, including in primary health services and hospitals has been expanded [56], other sites, such as NGOs targeting key populations in the epidemic such as MSM might be emphasized. To the extent that integration of HIV testing and counseling services in primary care has not been a success, programs might consider ways to make these services more targeted to the specific key populations most at risk from HIV.

## Supporting Information

S1 Appendix Detail of RDS. (DOCX)

S2 Appendix Dataset with Data Dictionary. (XLSX)

## References

[1] Jaffe HW, Valdiserri RO, De Cock KM. The reemerging HIV/AIDS epidemic in men who have sex with men. JAMA. 2007;298(20):2412–4. 18042919 10.1001/jama.298.20.2412

[2] Grulich AE, Kaldor JM. Trends in HIV incidence in homosexual men in developed countries. Sex Health. 2008;5(2):113–8. Epub 2008/07/01. . 18588775 10.1071/sh07075

[3] Baral S, Sifakis F, Peryskina A, Mogilnii V, Masenior NF, Sergeyev B, et al Risks for HIV infection among gay, bisexual, and other men who have sex with men in Moscow and St. Petersburg, Russia. AIDS Res Hum Retroviruses. 2012;28(8):874–9. Epub 2011/10/08. 10.1089/AID.2011.0264 . 21978380

[4] Sullivan PS, Carballo-Dieguez A, Coates T, Goodreau SM, McGowan I, Sanders EJ, et al Successes and challenges of HIV prevention in men who have sex with men. Lancet. 2012;380(9839):388–99. 10.1016/S0140-6736(12)60955-6 22819659 PMC3670988

[5] Baral S, Sifakis F, Cleghorn F, Beyrer C. Elevated risk for HIV infection among men who have sex with men in low- and middle-income countries 2000–2006: a systematic review. PLoS medicine. 2007;4(12):e339 Epub 2007/12/07. 10.1371/journal.pmed.0040339 18052602 PMC2100144

[6] van Griensven F, de Lind van Wijngaarden JW. A review of the epidemiology of HIV infection and prevention responses among MSM in Asia. AIDS. 2010;24 Suppl 3:S30–40. Epub 2010/10/15. 10.1097/01.aids.0000390087.22565.b400002030-201009003-00005 [pii]. . 20926926

[7] Copeland B, Shah B, Wheatley M, Heilpern K, del Rio C, Houry D. Diagnosing HIV in men who have sex with men: an emergency department's experience. AIDS patient care and STDs. 2012;26(4):202–7. Epub 2012/02/24. 10.1089/apc.2011.0303 22356726 PMC3317392

[8] Barbosa Júnior A, Szwarcwald CL, Pascom ARP, Souza PBd Júnior. Tendências da epidemia de AIDS entre subgrupos sob maior risco no Brasil, 1980–2004. Cadernos de saude publica. 2009;25:727–37. 19347198 10.1590/s0102-311x2009000400003

[9] Mello MA, Pinho AA, Tun W, Chinaglia M, Barbosa, Jr., D¡az J. Risky sexual behavior and HIV seroprevalence among MSM in Campinas, SP, Brazil. Campinas: Population Council, 2008 2008. Report No.

[10] Kerr LRFS, Mota RS, Kendall C, Pinho AdA, Mello MB, Guimarães MDC, et al HIV among MSM in a large middle-income country. Aids. 2013;27(3):427–35. 10.1097/QAD.0b013e32835ad504 23291540

[11] Brasil. Ministério da Saúde. Secretaria de Vigilância em Saúde. Departamento Nacional de DST/AIDS e Hepatites virais. Aids no Brasil: epidemia concentrada e estabilizada em populações de maior vulnerabilidade [AIDS in Brazil: concentrate epidemic in most vulnerable population] Brasília: Ministério da Saúde; 2012 [cited 2013 Ago 15].

[12] Marks G, Crepaz N, Senterfitt JW, Janssen RS. Meta-analysis of high-risk sexual behavior in persons aware and unaware they are infected with HIV in the United States: implications for HIV prevention programs. J Acquir Immune Defic Syndr. 2005;39(4):446–53. Epub 2005/07/13. . 16010168 10.1097/01.qai.0000151079.33935.79

[13] Abdool Karim SS, Abdool Karim Q, Gouws E, Baxter C. Global Epidemiology of HIV-AIDS. Infectious Disease Clinics of North America. 2007;21(1):1–17. 10.1016/j.idc.2007.01.010 17502227

[14] Anthropology and Epidemiology 2007; Dordrecht, Holland: D. Reidel.

[15] Lauby JL, Millett GA, LaPollo AB, Bond L, Murrill CS, Marks G. Sexual risk behaviors of HIV-positive, HIV-negative, and serostatus-unknown Black men who have sex with men and women. Archives of sexual behavior. 2008;37(5):708–19. Epub 2008/06/04. 10.1007/s10508-008-9365-6 . 18521734

[16] Flowers P, Knussen C, Li J, McDaid L. Has testing been normalized? An analysis of changes in barriers to HIV testing among men who have sex with men between 2000 and 2010 in Scotland, UK. HIV medicine. 2013;14(2):92–8. Epub 2012/09/01. 10.1111/j.1468-1293.2012.01041.x 22934820 PMC3561706

[17] Lyons A, Pitts M, Grierson J, Smith A, McNally S, Couch M. Sexual Behavior and HIV Testing Among Bisexual Men: A Nationwide Comparison of Australian Bisexual-Identifying and Gay-Identifying Men. AIDS Behav. 2012 Epub 2012/02/01. 10.1007/s10461-012-0148-7 . 22290610

[18] Marcus U, Hickson F, Weatherburn P, Schmidt AJ. Prevalence of HIV among MSM in Europe: comparison of self-reported diagnoses from a large scale internet survey and existing national estimates. BMC Public Health. 2012;12:978 Epub 2012/11/16. 10.1186/1471-2458-12-978 23151263 PMC3526585

[19] Hall HI, Walker F, Shah D, Belle E. Trends in HIV diagnoses and testing among U.S. adolescents and young adults. AIDS Behav. 2012;16(1):36–43. Epub 2011/04/13. 10.1007/s10461-011-9944-8 . 21484282

[20] Blas MM, Alva IE, Cabello R, Carcamo C, Kurth AE. Risk behaviors and reasons for not getting tested for HIV among men who have sex with men: an online survey in Peru. PLoS One. 2011;6(11):e27334 Epub 2011/11/19. 10.1371/journal.pone.0027334 22096551 PMC3212560

[21] Sherr L, Lopman B, Kakowa M, Dube S, Chawira G, Nyamukapa C, et al Voluntary counselling and testing: uptake, impact on sexual behaviour, and HIV incidence in a rural Zimbabwean cohort. AIDS. 2007;21(7):851–60. Epub 2007/04/07. 10.1097/QAD.0b013e32805e8711 . 17415040

[22] Lane T, Raymond HF, Dladla S, Rasethe J, Struthers H, McFarland W, et al High HIV prevalence among men who have sex with men in Soweto, South Africa: results from the Soweto Men's Study. AIDS Behav. 2011;15(3):626–34. Epub 2009/08/08. 10.1007/s10461-009-9598-y 19662523 PMC2888758

[23] Wagner GJ, Aunon FM, Kaplan RL, Rana Y, Khouri D, Tohme J, et al A qualitative exploration of sexual risk and HIV testing behaviors among men who have sex with men in Beirut, Lebanon. PLoS One. 2012;7(9):e45566 Epub 2012/10/03. 10.1371/journal.pone.0045566 23029103 PMC3445492

[24] Chow EP, Wilson DP, Zhang L. The rate of HIV testing is increasing among men who have sex with men in China. HIV Med. 2012;13(5):255–63. 10.1111/j.1468-1293.2011.00974.x . 22252151

[25] Zou H, Hu N, Xin Q, Beck J. HIV testing among men who have sex with men in China: a systematic review and meta-analysis. AIDS Behav. 2012;16(7):1717–28. 10.1007/s10461-012-0225-y . 22677975

[26] França Junior I, Calazans G, Zucchi EM. Mudanças no âmbito da testagem anti-HIV no Brasil entre 1998 e 2005. Revista de Saúde Pública. 2008;42:84–97. 18660928 10.1590/s0034-89102008000800011

[27] Brasil. Ministério da Saúde. Departamento de DST AeHV. Boletim Epidemiológico AIDS/DST. 2010.

[28] Do TD, Chen S, McFarland W, Secura GM, Behel SK, MacKellar DA, et al HIV testing patterns and unrecognized HIV infection among young Asian and Pacific Islander men who have sex with men in San Francisco. AIDS Educ Prev. 2005;17(6):540–54. Epub 2006/01/10. 10.1521/aeap.2005.17.6.540 . 16398576

[29] Sanchez T, Finlayson T, Drake A, Behel S, Cribbin M, Dinenno E, et al Human immunodeficiency virus (HIV) risk, prevention, and testing behaviors—United States, National HIV Behavioral Surveillance System: men who have sex with men, November 2003-April 2005. MMWR Surveill Summ. 2006;55(6):1–16. Epub 2006/07/11. . 16826162

[30] Holt M, Rawstorne P, Wilkinson J, Worth H, Bittman M, Kippax S. HIV testing, gay community involvement and internet use: social and behavioural correlates of HIV testing among Australian men who have sex with men. AIDS Behav. 2012;16(1):13–22. Epub 2011/01/08. 10.1007/s10461-010-9872-z . 21213035

[31] Oster AM, Miles IW, Le BCL, DiNenno EA, Wiegand RE, Heffelfinger JD, et al HIV Testing Among Men Who Have Sex with Men—21 Cities, United States, 2008. MMWR. 2011;60(21):6.

[32] Golden MR, Stekler J, Hughes JP, Wood RW. HIV Serosorting in Men Who Have Sex With Men: Is It Safe? JAIDS Journal of Acquired Immune Deficiency Syndromes. 2008;49(2):212–8 10.1097/QAI.0b013e31818455e8 18769346

[33] Sifakis F, Hylton JB, Flynn C, Solomon L, MacKellar DA, Valleroy LA, et al Prevalence of HIV infection and prior HIV testing among young men who have sex with men. The Baltimore young men's survey. AIDS and behavior. 2010;14(4):904–12. Epub 2007/10/31. 10.1007/s10461-007-9317-5 . 17968648

[34] Cohen MS, Chen YQ, McCauley M, Gamble T, Hosseinipour MC, Kumarasamy N, et al Prevention of HIV-1 infection with early antiretroviral therapy. The New England journal of medicine. 2011;365(6):493–505. Epub 2011/07/20. 10.1056/NEJMoa1105243 21767103 PMC3200068

[35] Branson BM. The future of HIV testing. J Acquir Immune Defic Syndr. 2010;55 Suppl 2:S102–5. Epub 2011/03/26. 10.1097/QAI.0b013e3181fbca44 . 21406978

[36] Heckathorn DD. Respondent-driven sampling: a new approach to the study of hidden populations. Social problems. 1997:174–99.

[37] Heckathorn DD. Extensions of Respondent Driven Sampling: Analyzing continuous variables and controlling for differential recruitment. Sociological Methodology. 2007;37(1):151–207.

[38] Johnston LG, Malekinejad M, Kendall C, Iuppa IM, Rutherford GW. Implementation challenges to using respondent-driven sampling methodology for HIV biological and behavioral surveillance: field experiences in international settings. AIDS Behav. 2008;12(4 Suppl):S131–41. 10.1007/s10461-008-9413-1 . 18535901

[39] Lansky A, Abdul-Quader AS, Cribbin M, Hall T, Finlayson TJ, Garfein RS, et al Developing an HIV behavioral surveillance system for injecting drug users: the National HIV Behavioral Surveillance System. Public Health Rep. 2007;122 Suppl 1:48–55. Epub 2007/03/16. 17354527 10.1177/00333549071220S108 PMC1804107

[40] Barbosa Junior A, Pascom AR, Szwarcwald CL, Kendall C, McFarland W. Transfer of sampling methods for studies on most-at-risk populations (MARPs) in Brazil. Cad Saude Publica. 2011;27 Suppl 1:S36–44. Epub 2011/04/29. . 21503522 10.1590/s0102-311x2011001300005

[41] ABEP [Brazilian Association of Research Companies]. Criterio de classificacao economica Brasil [Criteria of economic classification Brazil] Brazil: ABEP; 2008 [cited 2015 March 8, 2015]. Available from: http://www.abep.org/criterioBrasil.aspx.

[42] Szwarcwald CL, de Souza Junior PR, Damacena GN, Junior AB, Kendall C. Analysis of data collected by RDS among sex workers in 10 Brazilian cities, 2009: estimation of the prevalence of HIV, variance, and design effect. J Acquir Immune Defic Syndr. 2011;57 Suppl 3:S129–35. 10.1097/QAI.0b013e31821e9a36 . 21857308

[43] Wimonsate W, Naorat S, Varangrat A, Phanuphak P, Kanggarnrua K, McNicholl J, et al Factors associated with HIV testing history and returning for HIV test results among men who have sex with men in Thailand. AIDS Behav. 2011;15(4):693–701. Epub 2010/07/14. 10.1007/s10461-010-9755-3 . 20623251

[44] Brahmam GN, Kodavalla V, Rajkumar H, Rachakulla HK, Kallam S, Myakala SP, et al Sexual practices, HIV and sexually transmitted infections among self-identified men who have sex with men in four high HIV prevalence states of India. AIDS. 2008;22 Suppl 5:S45–57. Epub 2009/01/07. 10.1097/01.aids.0000343763.54831.15 . 19098479

[45] Neilands TB, Steward WT, Choi KH. Assessment of stigma towards homosexuality in China: a study of men who have sex with men. Arch Sex Behav. 2008;37(5):838–44. Epub 2008/02/16. 10.1007/s10508-007-9305-x . 18274889

[46] Vu BN, Girault P, Do BV, Colby D, Tran LT. Male sexuality in Vietnam: the case of male-to-male sex. Sex Health. 2008;5(1):83–8. Epub 2008/03/26. . 18361860 10.1071/sh07064

[47] Abell N, Rutledge SE, McCann TJ, Padmore J. Examining HIV/AIDS provider stigma: assessing regional concerns in the islands of the Eastern Caribbean. AIDS Care. 2007;19(2):242–7. Epub 2007/03/17. 10.1080/09540120600774297 . 17364405

[48] Lau JT, Lin C, Hao C, Wu X, Gu J. Public health challenges of the emerging HIV epidemic among men who have sex with men in China. Public Health. 2011;125(5):260–5. Epub 2011/06/11. 10.1016/j.puhe.2011.01.007 . 21658537

[49] Adam PC, de Wit JB, Toskin I, Mathers BM, Nashkhoev M, Zablotska I, et al Estimating levels of HIV testing, HIV prevention coverage, HIV knowledge, and condom use among men who have sex with men (MSM) in low-income and middle-income countries. Journal of acquired immune deficiency syndromes (1999). 2009;52 Suppl 2:S143–51. Epub 2009/12/05. 10.1097/QAI.0b013e3181baf11100126334-200912012-00010 [pii]. . 19901627

[50] de Arazoza H, Joanes J, Lounes R, Legeai C, Clemencon S, Perez J, et al The HIV/AIDS epidemic in Cuba: description and tentative explanation of its low HIV prevalence. BMC Infect Dis. 2007;7:130 Epub 2007/11/13. 10.1186/1471-2334-7-130 17996109 PMC2190762

[51] Knox J, Sandfort T, Yi H, Reddy V, Maimane S. Social vulnerability and HIV testing among South African men who have sex with men. Int J STD AIDS. 2011;22(12):709–13. Epub 2011/12/17. 10.1258/ijsa.2011.010350 22174050 PMC3243961

[52] Lo YC, Turabelidze G, Lin M, Friedberg Y. Prevalence and determinants of recent HIV testing among sexually active men who have sex with men in the St. Louis metropolitan area, Missouri, 2008. Sexually transmitted diseases. 2012;39(4):306–11. Epub 2012/03/17. 10.1097/OLQ.0b013e31824018d4 . 22421699

[53] Berg RC, Ross MW, Weatherburn P, Schmidt AJ. Structural and environmental factors are associated with internalised homonegativity in men who have sex with men: findings from the European MSM Internet Survey (EMIS) in 38 countries. Soc Sci Med. 2013;78:61–9. Epub 2012/12/25. 10.1016/j.socscimed.2012.11.033 . 23261257

[54] Ferreira MdPS, Silva CMFPd, Gomes MCF, Silva SMBd. Testagem sorológica para o HIV e a importância dos Centros de Testagem e Aconselhamento (CTA): resultados de uma pesquisa no município do Rio de Janeiro. Ciência & Saúde Coletiva. 2001;6:481–90.

[55] Grangeiro A, Escuder MM, Wolffenbüttel K, Pupo LR, Nemes MIB, Monteiro PHN. Avaliação do perfil tecnológico dos centros de testagem e aconselhamento para HIV no Brasil. Revista de Saúde Pública. 2009;43:427–36. 19448912 10.1590/s0034-89102009000300006

[56] Pinto RM, Wall M, Yu G, Penido C, Schmidt C. Primary care and public health services integration in Brazil's unified health system. American journal of public health. 2012;102(11):e69–76. 10.2105/AJPH.2012.300972 22994254 PMC3477957

