# Supplementary material for: Factors Associated with Low Levels of HIV Testing among Men Who Have Sex with Men (MSM) in Brazil
Source: PLoS One. 2015 Jun 22;10(6):e0130445. doi: 10.1371/journal.pone.0130445 (PMC4476690; doi:10.1371/journal.pone.0130445)
Supplement: S1 Appendix — (DOCX) [file pone.0130445.s001.docx]

**Appendix S1**

**S1 Table A. Reported network size, waves and sample size by city**

|  | # of seeds | Mean network size | Sd | Minimum | Maximum |
| --- | --- | --- | --- | --- | --- |
| **Reported individual network size** |  |  |  |  |  |
| Manaus | 10 | 40.10 | 91.810 | 2 | 300 |
| Recife | 10 | 11.30 | 14.591 | 3 | 50 |
| Salvador | 18 | 14.72 | 17.832 | 3 | 80 |
| Brasilia | 10 | 23.60 | 48.151 | 3 | 160 |
| Campo Grande | 7 | 19.29 | 9.759 | 5 | 30 |
| Belo Horizonte | 21 | 23.75 | 32.077 | 1 | 98 |
| Rio de Janeiro | 13 | 67.42 | 140.569 | 3 | 500 |
| Santos | 12 | 10.36 | 8.789 | 3 | 30 |
| Curitiba | 32 | 18.38 | 36.808 | 1 | 200 |
| Itajai | 15 | 12.07 | 10.965 | 3 | 40 |
| **Number of waves** |  |  |  |  |  |
| Manaus | 10 | 10.40 | 5.27 | 5 | 20 |
| Recife | 10 | 3.60 | 4.35 | 0 | 12 |
| Salvador | 18 | 2.83 | 5.22 | 0 | 20 |
| Brasilia | 10 | 5.70 | 5.23 | 0 | 17 |
| Campo Grande | 7 | 8.43 | 6.37 | 2 | 17 |
| Belo Horizonte | 21 | 2.52 | 3.83 | 0 | 15 |
| Rio de Janeiro | 13 | 3.00 | 3.49 | 0 | 12 |
| Santos | 12 | 2.50 | 4.46 | 0 | 16 |
| Curitiba | 32 | 2.09 | 3.23 | 0 | 13 |
| Itajai | 15 | 2.73 | 3.92 | 0 | 13 |
| **Number of participants recruited** |  |  |  |  |  |
| Manaus | 10 | 83.80 | 79.948 | 13 | 231 |
| Recife | 10 | 34.10 | 60.870 | 0 | 176 |
| Salvador | 18 | 20.28 | 56.183 | 0 | 235 |
| Brasilia | 10 | 33.40 | 53.583 | 0 | 177 |
| Campo Grande | 7 | 49.14 | 51.902 | 5 | 124 |
| Belo Horizonte | 21 | 12.05 | 29.162 | 0 | 128 |
| Rio de Janeiro | 13 | 26.46 | 69.303 | 0 | 255 |
| Santos | 12 | 24.33 | 75.220 | 0 | 263 |
| Curitiba | 32 | 9.53 | 31.460 | 0 | 178 |

**S1 Table B. Homophily and wave that equilibium was achieved for outcome variable ever tested for HIV**

| Parameter | Manaus | Recife | Salvador | Brasilia | Campo Grande | Belo Horizonte | Rio de Janeiro | Santos | Curitiba | Itajai |
| --- | --- | --- | --- | --- | --- | --- | --- | --- | --- | --- |
| **Homophily** |  |  |  |  |  |  |  |  |  |  |
| Negative | 0.001 | 0.169 | 0.071 | 0.165 | 0.054 | -0.061 | 0.322 | 0.187 | 0.046 | 0.056 |
| Positive | 0.189 | 0.295 | 0.240 | 0.203 | 0.098 | -0.034 | 0.493 | 0.139 | 0.277 | 0.351 |
| **Wave for equilibium** | 5 | 7 | 6 | 6 | 4 | 3 | 10 | 6 | 5 | 6 |
